# Descemet’s Stripping Automated Endothelial Keratoplasty (DSAEK) Management of Bullous Keratopathy After PreserFlo MicroShunt Device: A Case Report and Literature Review

**DOI:** 10.7759/cureus.81904

**Published:** 2025-04-08

**Authors:** Hideki Fukuoka, Morio Ueno, Minori Minamide, Chie Sotozono

**Affiliations:** 1 Department of Ophthalmology, Kyoto Prefectural University of Medicine, Kyoto, JPN; 2 Department of Ophthalmology, Baptist Eye Institute, Kyoto, JPN

**Keywords:** bullous keratopathy, dsaek, minimally invasive glaucoma surgery, preserflo microshunt, pseudoexfoliative glaucoma

## Abstract

Bullous keratopathy is a known complication following glaucoma filtration surgeries, and Descemet's stripping automated endothelial keratoplasty (DSAEK) has been reported as an effective management approach in several cases. However, management of bullous keratopathy in eyes with minimally invasive bleb surgery (MIBS) devices presents unique challenges. We report the first successful case of DSAEK in a patient with bullous keratopathy following PreserFlo MicroShunt (PMS) (Santen Pharmaceutical Co., Ltd., Osaka, Japan) implantation, with additional complications of prior vitrectomy for malignant glaucoma. The patient, a 69-year-old male, had undergone bilateral pseudoexfoliative glaucoma surgery, and subsequent DSAEK was performed, resulting in favorable postoperative outcomes despite the challenging presentation. This case demonstrates the feasibility of endothelial keratoplasty in eyes with PreserFlo devices and crucial insights for surgical management, including our modified technique with limited central Descemet's membrane stripping, shortening of the PMS device, and utilization of a thinner donor graft to overcome the unique challenges associated with glaucoma drainage devices.

## Introduction

The PreserFlo MicroShunt (PMS) (Santen Pharmaceutical Co., Ltd., Osaka, Japan) is a relatively recent minimally invasive bleb surgery (MIBS) device that has been developed for the treatment of open-angle glaucoma. The 8.5-mm SIBS (polystyrene-block-isobutylene-block-polystyrene) tube creates an alternative pathway for aqueous humor drainage from the anterior chamber to the subconjunctival space, effectively lowering intraocular pressure (IOP) while minimizing tissue disruption compared to traditional filtering surgery [1-3].

The PMS device has demonstrated encouraging efficacy and safety profiles, exhibiting reduced rates of hypotony in comparison to trabeculectomy. However, patients with glaucoma filtration devices remain susceptible to corneal endothelial cell loss and subsequent bullous keratopathy [4,5]. The management of bullous keratopathy in eyes with glaucoma drainage devices poses distinctive challenges, including preserving graft attachment in the context of potential IOP fluctuations and bleb-related complications.

Descemet's stripping automated endothelial keratoplasty (DSAEK) is preferred over penetrating keratoplasty for managing endothelial dysfunction due to its advantages, including faster visual recovery, lower astigmatism, decreased risk of graft rejection, and avoidance of open-sky condition [6]. There is limited literature on DSAEK outcomes in eyes with MIBS, particularly the PMS.

We present a case report of a patient with bullous keratopathy following PMS implantation and prior vitrectomy for malignant glaucoma who underwent DSAEK, the first such reported case. The surgical approach and postoperative outcomes are highlighted in this report.

## Case presentation

**Figure 1:**
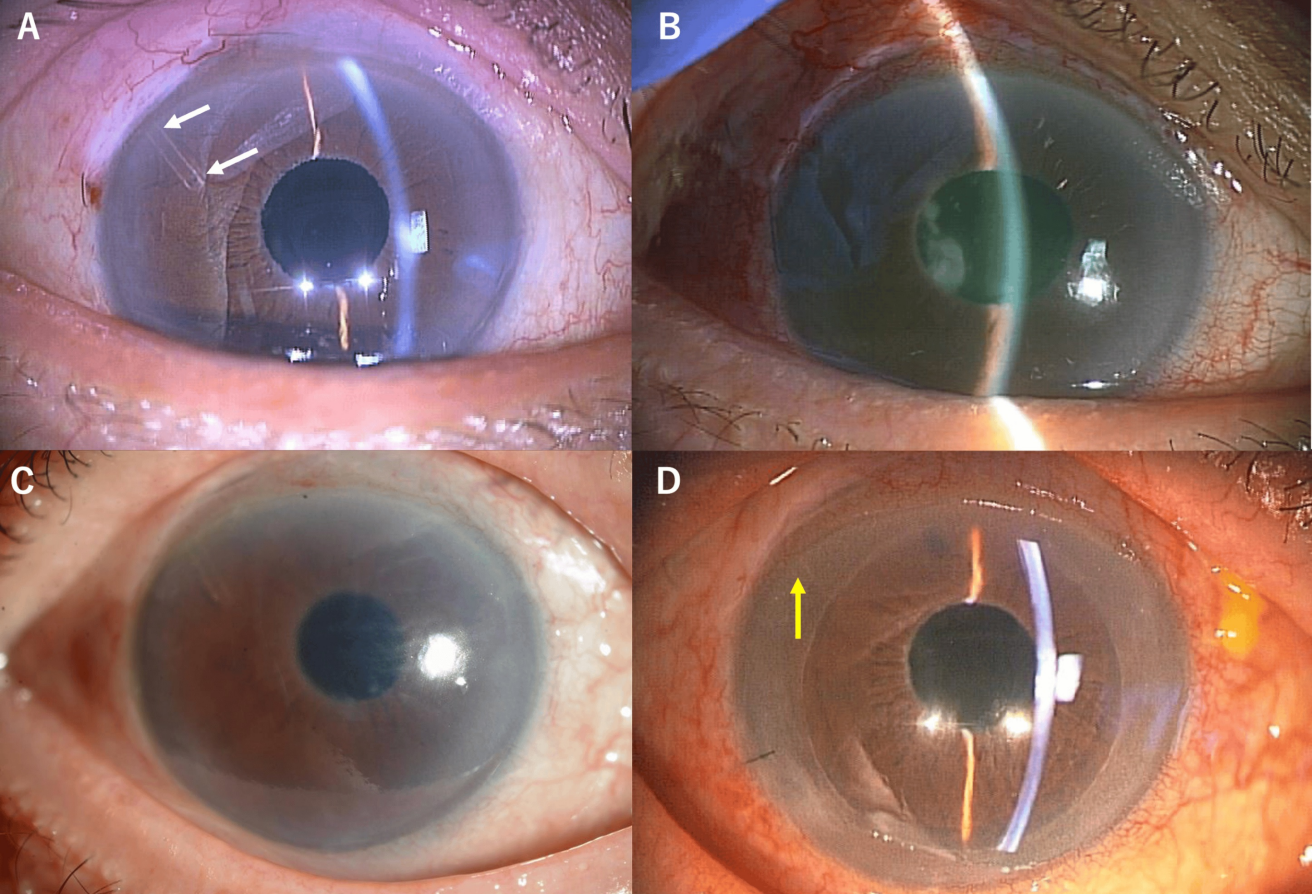
Clinical timeline of a patient with bullous keratopathy before and after Descemet's stripping automated endothelial keratoplasty (DSAEK) with PreserFlo MicroShunt (PMS). (A) Appearance of the right eye eight months after PMS implantation showing the PMS device visible at the 10 o'clock position (white arrows). No other tube shunts were implanted in this eye. (B) Image of the right eye showing malignant glaucoma with flattened anterior chamber following PMS implantation. (C) Preoperative slit-lamp image of the right eye obtained 19 months after PFM surgery reveals severe corneal edema, accompanied by the formation of bullae. (D) A photograph of the right eye demonstrating significant improvement in corneal clarity with well-positioned DSAEK graft and the shortened PMS device still visible (yellow arrow) post one-month DSAEK.

A 69-year-old male with bilateral pseudoexfoliative glaucoma and prior cataract surgery in his right eye was referred to our cornea specialty clinic with bullous keratopathy secondary to multiple factors in his right eye. The patient had no significant systemic comorbidities, including no history of diabetes, hypertension, or autoimmune diseases that could increase the risk of graft rejection. His ocular history was significant for multiple glaucoma surgeries in the right eye, including a trabeculotomy performed approximately three years ago (35 months) and PMS implantation 19 months prior to presentation. Prior to PMS implantation, endothelial cell count was already low at 1,149 cells/mm², likely due to pseudoexfoliative glaucoma, and decreased further to 997 cells/mm² at three months post-implantation. The patient's condition remained stable (Figure 1A) until the bleb reconstruction surgery, which was performed 5.5 months prior to presentation. Subsequently, malignant glaucoma developed in the right eye two weeks later (five months prior to presentation) (Figure 1B), a rare but reported complication of PMS implantation [7], which required pars plana vitrectomy (PPV) with irido-zonulo-hyaloid vitrectomy, resulting in a vitrectomized eye. The patient reported noticing a decline in vision in his right eye beginning one month prior to referral to our clinic.

At presentation, his visual acuity was hand motion, and IOP was 10 mmHg in the right eye. Slit-lamp examination revealed significant corneal edema with bullae formation in the right eye (Figure 1C). Endothelial cell density (ECD) could not be measured with non-contact specular microscopy due to severe corneal edema. Central corneal thickness was markedly increased at 739 μm as measured by anterior segment optical coherence tomography (Casia 2; Tomey Corporation, Nagoya, Japan).

After a thorough discussion of treatment options, DSAEK was planned for the right eye. The donor cornea had an ECD of 2,625 cells/mm² with a graft thickness of 68 μm. The surgery was performed using an 8 mm diameter pre-cut donor graft. We performed limited central Descemet's membrane stripping over a 5-6 mm diameter area, which is one step smaller than the pre-cut donor graft diameter to prevent unexpected detachment or folding of Descemet's membrane specifically at the peripheral adhesion zone of the graft, which could otherwise inhibit proper adhesion. We used a pre-cut donor graft from an eye bank and inserted it using the Busin glide technique. During surgery, approximately 1 mm of the PMS device was cut and removed to shorten it. Prior to air injection, balanced salt solution (BSS) was thoroughly infused through the anterior chamber into the vitreous cavity to ensure adequate posterior support. Air was injected into the anterior chamber using a 32-gauge needle to elevate IOP for graft attachment; however, we observed a tendency for IOP to decrease after several minutes. The air may have escaped into the bleb through the PMS or posteriorly into the vitreous cavity, which could contribute to the observed difficulty in maintaining adequate pressure for graft attachment (Video 1).

**Video 1:** Modified DSAEK technique for eye with Preserflo MicroShunt device DSAEK: Descemet's stripping automated endothelial keratoplasty

Aqueous humor samples obtained from the anterior chamber were all negative for Herpes Simplex Virus, Varicella-Zoster Virus, and Cytomegalovirus by polymerase chain reaction (PCR) analysis. Postoperatively, the patient was positioned in a supine position. Upon examination three hours after surgery, most of the air had disappeared from the anterior chamber, but the graft demonstrated good adhesion to the host cornea. The patient was discharged a couple of days after surgery.

**Figure 2:**
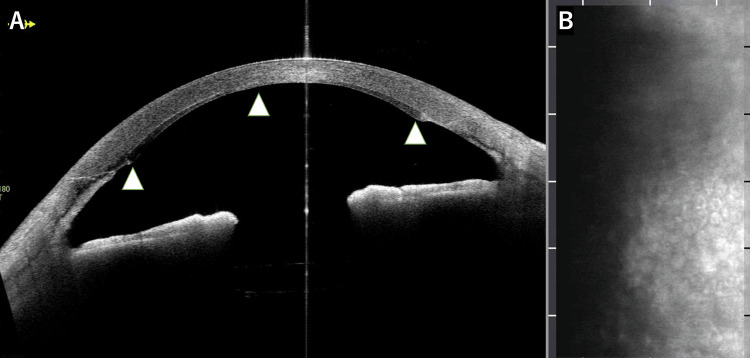
One-month postoperative imaging of the right eye. (A) Anterior segment optical coherence tomography demonstrating well-attached DSAEK graft with appropriate thickness (white arrowheads) and (B) specular microscopy revealing the presence of small endothelial cells, though automated cell counting was not feasible at this stage of recovery.

At the one-month postoperative visit, the patient's visual acuity had improved to 0.4 (decimal), the graft remained well-attached (Figures 1D, 2A), and the corneal clarity had improved significantly, allowing for successful specular microscopy imaging of endothelial cells (Figure 2B), and the PMS device continued to function properly with adequate IOP control at 10 mmHg without any glaucoma medications. No early postoperative complications, such as increased IOP, pupillary block, hypotony, or graft detachment, etc., were observed during the follow-up period.

## Discussion

This case demonstrates that DSAEK can be successfully performed in eyes with PMS devices, despite the challenges posed by the presence of a functioning filtration device and the absence of vitreous support. To the best of our knowledge, this is the first reported case of DSAEK in a patient with a PMS device.

Several considerations made this case particularly challenging. First, the absence of vitreous support in a vitrectomized eye generally elevates the risk of posterior graft dislocation [8]. Second, the functional PMS device engenders continuous aqueous outflow, thereby complicating the maintenance of adequate air tamponade for graft attachment. Third, the potential for postoperative IOP fluctuations in an eye with a glaucoma drainage device poses a risk of both graft detachment with hypotony and endothelial cell damage with elevated IOP.

The surgical approach was adapted in several ways to address these challenges. We performed limited central Descemet's membrane stripping which is one step smaller than the pre-cut donor graft diameter, to prevent unexpected detachment or folding of Descemet's membrane specifically in the peripheral adhesion zone of the graft, which could otherwise inhibit proper adhesion. Additionally, we reduced the length of the PMS device by approximately 1 mm to mitigate any potential interference with the graft. The adhesion properties of the graft were enhanced by employing a thinner donor graft. Thorough infusion of BSS into the vitreous cavity was performed to create adequate posterior pressure, which effectively prevented air migration behind the iris through the peripheral iridectomy (PI) incision. To mitigate trauma, a gentle air injection technique with a 32-gauge needle was employed. Postoperatively, despite the rapid evacuation of anterior chamber air, we maintained prolonged supine positioning to promote graft attachment (Table 1).

**Table 1:** Challenges, Contributing Factors, and Management Strategies in DSAEK with PreserFlo MicroShunt (PMS)

Challenge	Contributing Factor	Management Strategy
Air Management and Intraocular Pressure (IOP) Control		
Compromised air tamponade sustainability	Continuous aqueous and air outflow through PMS device	Use of 32G needle for precise and gentle air injection to minimize leakage; Prolonged supine positioning postoperatively
Posterior air migration	Vitrectomized eye without adequate posterior pressure	Infusion of BSS into vitreous cavity to create adequate posterior pressure and prevent iris elevation toward cornea, which would complicate surgery
Pupillary block glaucoma	Excessive air in anterior chamber	Creation of appropriate peripheral iridectomy during surgery
Postoperative intraocular pressure dysregulation	Functioning filtration device	Mandatory examination at 3 hours post-surgery; Air removal through surgical port if pupillary block occurs or if IOP is excessively high
Graft Attachment		
Graft detachment	Low IOP due to air evacuation through PMS	If graft detachment is observed at 3-hour check due to low IOP, consider additional air injection or graft suturing
Peripheral graft non-adherence	Inadequate peripheral adhesion	Limited central Descemet's membrane stripping one step smaller than graft diameter; Use of thinner donor graft to enhance adhesion properties
Mechanical interface disturbance	Physical presence of the PMS device	Shortening of PMS device to prevent interference with graft positioning
Inadequate graft adherence	Thick donor tissue	Preference for thinner donor grafts to enhance adhesion properties

Our comprehensive literature search included not only major indexed databases (PubMed, Embase, Scopus, and Google Scholar) but also extended to conference proceedings, non-indexed journals, and grey literature through April 2, 2025, confirming that this case represents the first documented instance of DSAEK in a patient with a PMS device. This systematic search, employing a combination of terms including “DSAEK,” “Descemet's stripping automated endothelial keratoplasty,” and “PreserFlo,” confirmed that our case represents the first documented instance of this specific surgical sequence. Although there are studies of endothelial keratoplasty in eyes with other glaucoma drainage devices, they typically report a higher rate of complications, including corneal endothelial cell loss, graft detachment, failure, and secondary glaucoma [9,10].

The extant data indicate that PMS demonstrates a generally favorable safety profile with respect to corneal ECD. A multitude of studies have demonstrated that there is no significant reduction in ECD at 12 months post-implantation when compared to non-implanted contralateral eyes [11,12]. Nevertheless, there are long-term concerns, as articulated by Chamard et al. who documented cases of endothelial cell loss at five years post-implantation [13]. The proposed mechanisms underlying this variability include direct mechanical trauma during device insertion, chronic inflammation, or altered aqueous humor dynamics affecting nutrient delivery to endothelial cells.

In this case, the positive outcome observed one month after DSAEK, accompanied by a substantial enhancement in visual acuity and corneal clarity, suggests that endothelial keratoplasty may be a viable treatment option for bullous keratopathy in eyes equipped with PMS devices. The rapid clearance of air from the anterior chamber demonstrates the continuous filtration by the device. However, this did not prevent graft adherence, which may be attributed to the modified surgical approach and careful postoperative management employed in this study.

## Conclusions

This case report demonstrates the successful performance of DSAEK in eyes with PMS devices, even in the challenging setting of a vitrectomized eye. Our experience suggests that, with appropriate surgical modifications and careful postoperative management, positive outcomes can be achieved. As the utilization of MIBS devices continues to expand, it is imperative that corneal surgeons be cognizant of the potential challenges and adaptations necessary when performing endothelial keratoplasty in these eyes. Further studies with larger cohorts and longer follow-up are needed to determine the long-term outcomes and optimal surgical approaches for endothelial keratoplasty in eyes with MIBS devices.
